# Burden of psychiatric disease inversely correlates with Alzheimer's age at onset

**DOI:** 10.1002/alz.70677

**Published:** 2025-10-23

**Authors:** Emily Eijansantos, Isabel E. Allen, Jessica de Leon, Stephanie Grasso, Nicole Rogers, Rian Bogley, Andrew Paramo, Alexander J. Ehrenberg, Maxime Montembeault, Virginia Sturm, Salvatore Spina, Lea T. Grinberg, William W. Seeley, Katherine P. Rankin, Joel H. Kramer, Howard J. Rosen, Gil D. Rabinovici, Maria Luisa Gorno‐Tempini, Bruce L. Miller, David C. Perry, Zachary A. Miller

**Affiliations:** ^1^ Department of Neurology University of California San Francisco California USA; ^2^ Department of Biostatistics University of California San Francisco California USA; ^3^ Memory and Aging Center, Department of Neurology, Weill Institute for Neurosciences University of California San Francisco California USA; ^4^ Department of Speech, Language, and Hearing Sciences University of Texas Austin Texas USA; ^5^ Global Brain Health Institute University of California San Francisco California USA; ^6^ Dyslexia Center, Department of Neurology and Psychiatry, UCSF Weill Institute for Neurosciences University of California San Francisco California USA; ^7^ Department of Psychiatry NSU Orlando VA Healthcare System Orlando Florida USA; ^8^ Department of Psychiatry McGill University & Douglas Research Centre Verdun QC Canada; ^9^ Department of Pathology University of California San Francisco California USA

**Keywords:** Alzheimer's disease, anxiety, bipolar disorder, depression, early onset Alzheimer's disease, post‐traumatic stress disorder, psychiatric disease, schizophrenia

## Abstract

**INTRODUCTION:**

Depression is regarded as a risk factor for Alzheimer's disease (AD). Associations between AD and other psychiatric disorders are less clear.

**METHODS:**

We screened 1,500 AD UCSF Memory and Aging Center patients for prevalence of psychiatric disorders and compared results to 8,267 NACC AD participants.

**RESULTS:**

AD with depression, anxiety, or post‐traumatic stress disorder were significantly younger at age at onset than AD without (*p *< 0.001; *p *< 0.001; *p *< 0.05). Comorbidity of depression, anxiety and PTSD led to further decreases in AD age at onset. Within the NACC cohort, we further demonstrated an inverse relationship between the severity of depression and anxiety symptoms and AD age at onset.

**DISCUSSION:**

Depression, anxiety, and post‐traumatic stress disorder are inversely associated with AD age at onset. Age at onset further decreases with increasing number of psychiatric conditions and increasing severity of symptoms, suggesting that overall burden of psychiatric disease is highly relevant to AD.

**Highlights:**

- Retrospective chart review revealed that in patients with AD, those who also had depression, anxiety, or post‐traumatic stress disorder were significantly younger at age at onset than those without.
- Increasing burden of psychiatric disease, both in severity of psychiatric symptoms and number of comorbid psychiatric conditions, produced serial decreases in the age at onset of AD.
- In patients with AD, those with depression were more likely to have autoimmune disease, and those with anxiety were more likely to have a history of seizures.

## 1 INTRODUCTION

Well‐established, typical risk factors for Alzheimer's disease (AD) can be organized into several broad categories, such as vascular disease, head trauma, insufficient cognitive stimulation, and mental health conditions.^1^, ^2^, ^3^ Improving conditions in many of these risk factors (e.g., awareness of healthy diets, modern medications for reducing hypertension and hyperlipidemia, improved access to education) has led to decreases in AD incidence.^4^, ^5^ In contrast, there is an epidemic of depression, social isolation, and poor mental health among aging populations,^6^, ^7^ and thus there is palpable urgency to better understand how mental health conditions interact with AD. While depression has been heavily discussed as both a risk factor for and prodromal symptom of AD, the impacts of other prominent psychiatric disorders including anxiety,^8^, ^9^, ^10^, ^11^ post‐traumatic stress disorder (PTSD),^12^, ^13^, ^14^, ^15^, ^16^, ^17^ bipolar disorder (BPD),^18^, ^19^, ^20^, ^21^ and schizophrenia (SCZ)^22^, ^23^ are less established. Beyond this, only a handful of studies have attempted to detail the impact that comorbid psychiatric conditions have on AD.^10^, ^24^, ^25^ Further, it is actively debated as to whether mental health conditions reflect risk factors for AD or instead represent a prodrome of AD itself. Previously, we observed inverse relationships between the novel factors (non‐right‐handedness, neurodevelopmental disorders, autoimmune disease, and seizures) and age at AD symptom onset in a large, heterogenous cohort of 1,500 Alzheimer's disease cases from the UCSF Memory and Aging Center (MAC), which consisted of half early onset AD (EOAD, first symptoms < 65) and half late onset AD (LOAD) and included non‐amnestic forms of AD [logopenic variant primary progressive aphasia (lvPPA) and posterior cortical atrophy (PCA)].^26^ Here we sought to describe the prevalence and timing of a wide range of psychiatric conditions across this cohort, with a focus on AD age at onset, and then employed collections from the National Alzheimer's Coordinating Center (NACC) cohort as a means of replicating our findings.

## 2 METHODS

### 2.1 Cohorts

The UCSF MAC cohort consisted of 750 EOAD and 750 LOAD subjects who met diagnostic (NINCDS‐ADRDA and later NIA‐AA) criteria for probable AD evaluated at the UCSF MAC from 1998‐2016. The NACC cohort was used as an external replication cohort (with UCSF MAC cases excluded to avoid duplication). Details of the selection criteria for AD individuals from the NACC database can be found in our prior publication.^26^

### 2.2 Identification and classification of psychiatric disorders

Charts were reviewed in a retrospective manner, screening for patient report of depression, anxiety, PTSD, BPD, and SCZ in the patient's first visit note from the UCSF MAC. The UCSF MAC History and Exam consists of a semi‐structured interview between the clinician, the participant, and their informant. As such, capture of psychiatric symptoms from chart review came from two sources, either 1) report of a psychiatric disease from an outside clinician, an “external clinician diagnosis,” or 2) by direct report of psychiatric symptoms by the patient or caregiver to the evaluating MAC clinician, “internal MAC clinician report.” Age at first cognitive symptom and age at first psychiatric symptom were extracted from these visit notes. From these ages, we binned the data into a total of seven categories: 1) those with unknown onset of psychiatric symptoms, 2) those with onset of psychiatric symptoms after first cognitive symptoms, those with onset of psychiatric symptoms within a period of 3) 0‐10 years, 4) 11‐20 years, 5) 21‐30 years, 6) 31‐40 years, and 7) greater than 40 years prior to onset of cognitive symptoms. While conventional attitudes about depression within bipolar disorder suggest that bipolar mood disorder subsumes the diagnosis of depression,^27^ this remains an active area of debate,^28^ with some arguing that depression should carry independent notation within the context of bipolar disease. As such, in this study, bipolar disorder and depression in individuals with both were counted as separate entities to be consistent with how they were described in the patient's chart and to distinguish different phenotypes of psychiatric disease, given our interest in detailing the additive effects of psychiatric symptoms on AD age at onset and that not all BPD cases had a history of depressive episodes. Cases were also screened for other novel factors as previously reported^26^ including non‐right‐handedness, autoimmune disease, history of seizures, and developmental differences.

### 2.3 NACC As A Replication Cohort With An Orthogonal Measure Of Psychiatric Symptoms

While the most recent iterations of the NACC database (version 3) standardly collect physician history of depression, anxiety, PTSD, bipolar disease, and schizophrenia, the cohort we investigated had only a limited number of subjects with version 3 (*n* = 196). Within version 1.2 and 2 of the NACC, information about anxiety is only collected within the Neuropsychiatric Inventory Questionnaire (NPI‐Q). Thus, we relied exclusively on the NPI‐Q assessment in this study, restricted to the domains of anxiety and depression. In the NACC cohort, 8,267 patients had NPI‐Q data, which in addition to capturing the presence or absence of symptoms, also included caregiver estimates of the severity of depression and anxiety experienced by each patient.^29^ The NPI‐Q measures severity of symptoms by this caregiver report within the domains of depression and anxiety in which the options were absent, mild, moderate, or severe. The NPI‐Q captures current ratings of severity of depression and anxiety at the subject's initial visit, thus it does not capture symptom onset and as such, categories based on the timing of psychiatric symptom onset do not apply for the NACC cohort.

### 2.4 Data analyses

For comparisons between groups, Analysis of Variance (ANOVA) or Students Independent group t‐tests were used for continuous variables such as age at onset and years of education, and Fisher's exact or Chi‐squared tests were used for nominal variables such as sex and non‐right‐handedness. Odds ratios for risk factors were calculated using logistic regression. All analyses were performed with STATA 18.1 with significance levels set to *p *< 0.05.

## 3 RESULTS

Within the UCSF MAC cohort, 43.3% (652/1500) of patients had a history of depression, 32.3% (485/1500) anxiety, 1.2% (18/1500) BPD, 1% (15/1500) PTSD, and 0.4% (6/1500) SCZ. Statistically higher amounts of depression (49.1% vs. 38.0%, *p *< 0.001) and anxiety (39.7% vs. 24.9%, *p *< 0.001), were observed in EOAD compared to LOAD, an increased trend was observed in PTSD (1.5% vs. 0.5%, *p *= 0.069), whereas rates were no different for BPD (1.2% vs. 1.2%) and SCZ (0.5% vs. 0.3%). Logistic regressions performed to predict the different rates of psychiatric conditions in EOAD vs. LOAD, controlling for sex, education, *APOE* ɛ4 status, and typical AD risk factors (hypertension, hypercholesteremia, diabetes), confirmed all significant direct comparisons (Table 1). Comparisons between amnestic vs. non‐amnestic AD failed to show any differences in rates of psychiatric disorders. Breaking down non‐amnestic AD into the constituents of lvPPA and PCA, PCA showed more depression (48.7% vs. 36.8%, *p *= 0.048, Supplemental Table 1).

1 **RESEARCH IN CONTEXT**

1. **Systematic review**: The authors used Pubmed to review literature pertaining to Alzheimer's disease (AD) and psychiatric conditions: depression, anxiety, bipolar disorder, post‐traumatic stress disorder (PTSD), and schizophrenia. There is abundant literature exploring the associations between depression and AD and increasing literature surrounding anxiety and AD. However, studies related to PTSD, bipolar disorder, and schizophrenia and AD are limited, and investigations of the comorbidity of multiple psychiatric conditions and AD are sparse.
2. **Interpretation**: The prevalence of depression, anxiety, and PTSD are each inversely associated with AD age at onset. Age at onset further decreases with increasing number of psychiatric conditions as well as increasing severity of symptoms of depression and anxiety.
3. **Future directions**: Further work needs to be done to delineate the mechanisms by which psychiatric conditions influence and are influenced by AD pathophysiology.

**TABLE 1:** Psychiatric Disorders in EOAD vs. LOAD.

	UCSF MAC 1,500 AD		
Group Demographics	EOAD (*n* = 750)	LOAD (*n* = 750)	*p*
**Age at onset**			
Average years ± Std	**55.8 ± 5.5**	**71.5 ± 4.2**	**<0.001**
**Age at first visit**			
Average years ± Std	**60.2 ± 5.9**	**75.0 ± 4.3**	**<0.001**
**Sex**			
% Male (*n*)	41.9% (314/750)	44.1% (331/750)	0.375
**Education**			
Average years ± Std	15.2 ± 3.6 (728)	15.1 ± 3.8 (729)	0.464
***APOE* ɛ4 carriers**	**52.5% (156/297)**	**63.0% (102/162)**	**0.031**

**Psychiatric Disorders**			
**Depression**			
% Depression (*n*)	**49.1% (368/750)**	**38.0% (285/750)**	**<0.001**
**Anxiety**			
% Anxiety (*n*)	**39.7% (298/750)**	**24.9% (187/750)**	**<0.001**
**PTSD**			
% PTSD (*n*)	1.5% (11/750)	0.5% (4/750)	0.069
**BPD**			
% Bipolar Disorder (*n*)	1.2% (9/750)	1.2% (9/750)	1
**SCZ**			
% Schizophrenia (*n*)	0.5% (4/750)	0.3% (2/750)	0.414

	95% C.I. for OR			
Increased EOAD Prevalence	OR	Lower	Upper	*p*
**Depression**	**1.984**	**1.280**	**1.931**	**<0.001**
**Anxiety**	**1.984**	**1.592**	**2.475**	**<0.001**
**PTSD**	2.793	0.885	8.849	0.080
**BPD**	1.005	0.397	2.545	0.991
**SCZ**	2.008	0.367	10.989	0.422

Abbreviations: AD, Alzheimer's disease; BPD, Bipolar disorder; C.I., confidence interval; EOAD, early‐onset Alzheimer's disease; LOAD, late‐onset Alzheimer's disease; OR, odds ratio; PTSD, Post‐traumatic stress disorder; SCZ, Schizophrenia; Std, standard deviation.

Within the UCSF MAC cohort, those with depression, anxiety, or PTSD were 2.2, 3.0, and 6.8 years younger at age at onset of AD symptoms, respectively, compared to those without (depression *p *< 0.001; anxiety *p *< 0.001; PTSD *p *< 0.05). These results were no different when comparing “external diagnosis” to “internal report” of psychiatric disease (Supplemental Table 2) (of note this only pertained to diagnoses of depression and anxiety as all PTSD diagnoses came from a formal “external diagnosis”). Groups with depression or anxiety were more female than those without (*p *< 0.001) and possessed less hypertension, type 2 diabetes, and hyperlipidemia (*p *< 0.05 for each) (Table 2). Within the NACC cohort, those with current symptoms of depression and those with current symptoms of anxiety were each 2.1 years younger at AD age at onset than those without (*p *< 0.001 for each) (Supplemental Table 3).

**TABLE 2:** Interactions between Psychiatric Disorders with Typical and Novel AD Risk Factors within UCSF MAC.

Group	No Depression	All Depression	No Anxiety	All Anxiety	No PTSD	PTSD	No BPD	BPD	No SCZ	SCZ
Demographics	(*n* = 848)	(*n* = 652)	(*n* = 1015)	(*n* = 485)	(*n* = 1485)	(*n* = 15)	(*n* = 1482)	(*n* = 18)	(*n* = 1494)	(*n* = 6)
**Age at onset**										
Average years ± Std	**64.6 ± 9.2** ^**^	**62.4 ± 9.2** ^**^	**64.6 ± 9.1** ^**^	**61.6 ± 9.2** ^**^	**63.7 ± 9.2** ^*^	**56.9 ± 11.1** ^*^	63.7 ± 9.3	63.5 ± 7.6	63.7 ± 9.3	61.0 ± 9.4
**Age at first visit**										
Average years ± Std	**68.5 ± 9.1** ^**^	**66.5 ± 8.8** ^**^	**68.6 ± 8.9** ^**^	**65.6 ± 8.9** ^**^	**67.7 ± 9.0** ^*^	**61.4 ± 9.7** ^*^	67.6 ± 9.0	67.6 ± 7.9	67.6 ± 9.0	68.5 ± 6.6
**Sex**	**48.4%** ^**^	**36.1%** ^**^	**47.6%** ^**^	**33.4%** ^**^	43.1%	33.3%	42.8%	55.6%	43.0%	33.3%
% Male (*n*)	**(409/848)**	**(236/652)**	**(483/1015)**	**(162/485)**	(640/1485)	(5/15)	(635/1482)	(10/18)	(643/1494)	(2/6)

Typical Risk Factors										
**Education**	15.2 ± 3.6	15.1 ± 3.8	15.1 ± 3.8	15.3 ± 3.5	15.2 ± 3.7	16.3 ± 3.0	15.2 ± 3.7	15.3 ± 2.3	15.2 ± 3.7	15.2 ± 2.9
Average years ± Std	(825)	(632)	(990)	(467)	(1442)	(15)	(1439)	(18)	(1451)	(6)
***APOE* ɛ4 carriers**	54.1% (140/259)	59% (118/200)	56.9% (177/311)	54.7% (81/148)	56.6% (258/456)	0.0% (0/3)	56.2% (257/457)	50% (1/2)	56.1% (257/458)	100% (1/1)
**Hypertension**	**47.5%** ^*^	**41.3%** ^*^	**47.2%** ^*^	**39.8%** ^*^	44.9%	33.3%	44.9%	38.9%	44.7%	66.7%
% HTN (*n*)	**(403/848)**	**(269/652)**	**(479/1015)**	**(193/485)**	(667/1485)	(5/15)	(665/1482)	(7/18)	(668/1494)	(4/6)
**Hyperlipidemia**	**50.8%** ^*^	**45.2%** ^*^	**50.9%** ^*^	**42.9%** ^*^	48.6%	20%	48.3%	50.0%	48.4%	33.3%
% HLD (*n*)	**(431/848)**	**(294/652)**	**(517/1015)**	**(208/485)**	(722/1485)	(3/15)	(716/1482)	(9/18)	(723/1494)	(2/6)
**Diabetes**	**12.4%** ^*^	**9.0%** ^*^	**13.0%** ^*^	**6.6%** ^**^	11.0%	0.0%	11.0%	5.6%	10.9%	16.7%
% DM (*n*)	**(105/848)**	**(59/652)**	**(132/1015)**	**(32/485)**	(164/1485)	(0/15)	(163/1482)	(1/18)	(163/1495)	(1/6)

**Novel Factors**										
**non‐Right‐Handed**	12.3%	11.5%	12.0%	11.8%	12.0%	6.7%	11.9%	16.7%	11.9%	16.7%
% nRH (*n*)	(104/848)	(75/652)	(122/1015)	(57/485)	(178/1485)	(1/15)	(176/1482)	(3/18)	(178/1494)	(1/6)
**Learning Disability**	5.9%	7.8%	6.0%	8.2%	6.7%	13.3%	6.7%	5.6%	6.8%	0.0%
% LD (*n*)	(50/848)	(51/652)	(61/1015)	(40/485)	(991485)	(2/15)	(100/1482)	(1/18)	(101/1494)	(0/6)
**Seizure**	10.7%	7.4%	**5.3%** ^*^	**9.5%** ^*^	6.6%	13.3%	6.7%	0%	6.7%	0.0%
% Seizure (*n*)	(52/848)	(48/652)	**(54/1015)**	**(46/485)**	(98/1485)	(2/15)	(100/1482)	(0/18)	(100/1494)	(0/6)
**Autoimmune Disease**	**20.2%** ^*^	**25.6%** ^*^	22.3%	23.1%	22.5%	26.7%	22.4%	33.3%	22.6%	0.0%
% Autoimmunity (*n*)	**(171/848)**	**(167/652)**	(226/1015)	(112/485)	(334/1485)	(4/15)	(332/1482)	(6/18)	(338/1494)	(0/6)

Psych Dz										
**Depression**			**30.0%** ^**^	**71.2%** ^**^	**43.0%** ^**^	**93.3%** ^**^	43.1%	77.8%	43.5%	50.0%
% Depression (*n*)			**(304/1015)**	**(349/485)**	**(639/1485)**	**(14/15)**	(638/1482)	(14/18)	(650/1494)	(3/6)
**Anxiety**	**16.1%** ^**^	**53.4%** ^**^			**31.7%** ^**^	**100%** ^**^	32.2%	44.4%	32.3%	33.3%
% Anxiety (*n*)	**(136/848)**	**(349/652)**			**(470/1485)**	**(15/15)**	(477/1482)	(8/18)	(483/1494)	(2/6)
**PTSD**	0.1%	2.1%	0.0%	3.1%			0.9%	5.6%	1.0%	0.0%
% PTSD (*n*)	(1/848)	(14/652)	(0/1015)	(15/485)			(14/1482)	(1/18)	(15/1494)	(0/6)
**BPD**	0.5%	2.1%	1.0%	1.6%	1.1%	6.7%			1.2%	0.0%
% Bipolar Disorder (*n*)	(4/848)	(14/652)	(10/1015)	(8/485)	(17/1485)	(1/15)			(18/1494)	(0/6)
**SCZ**	0.4%	0.5%	0.4%	0.4%	0.4%	0%	0.4%	0%		
% Schizophrenia (*n*)	(3/848)	(3/652)	(4/1015)	(2/485)	(6/1485)	(0/15)	(6/1482)	(0/18)		

Abbreviations: AD, Alzheimer's disease; DPN, Depression; PTSD, Post‐traumatic stress disorder; BPD, Bipolar disorder; SCZ, Schizophrenia; Std, standard deviation.

* *p* < 0.05.

** *p* < 0.001.

To investigate if the timing of psychiatric symptoms impacted AD age at onset, we further divided the UCSF MAC cohort into seven categories organized by psychiatric symptom onset relative to AD age onset (as described in the Methods Section 2). There were no significant differences in AD age at onset between those with depression and anxiety when comparing all categories of psychiatric symptoms prior to AD age at onset or when comparing all groups together (Supplemental Table 4a and 4b).

**FIGURE 1:**
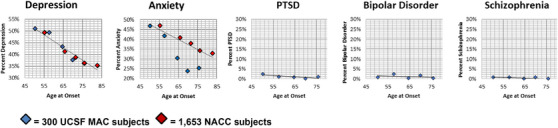
Distribution of Psychiatric Disorders Across the Range of Age at Onset. Each diamond represents a quintile with its respective cohort, with each blue diamond reflecting the 300 UCSF MAC subjects and each red diamond reflecting 1,653 NACC subjects who had NPI‐Q data (except for the oldest quintile of the NACC cohort, which represents 1,655 subjects). Each diamond is plotted out with the average of that specific cohort by the percent with a specific psychiatric condition, like depression. The exact numerical values for each quintile can be found in supplemental material (Supplemental Tables 5a and 5b). Best‐fit lines were generated that accounted for weighted differences between the UCSF MAC and NACC cohorts. Age at Onset, Age at first symptom of Alzheimer's disease; PTSD, Post‐traumatic stress disorder.

Rates of psychiatric disease were plotted by quintiles of AD age at onset in both UCSF MAC and NACC cohorts (Figure 1). Rates of depression, anxiety, and PTSD demonstrated significant inverse linear relationships with AD age at onset, while BPD and SCZ showed no differences (Supplemental Table 5a and 5b).

**FIGURE 2:**
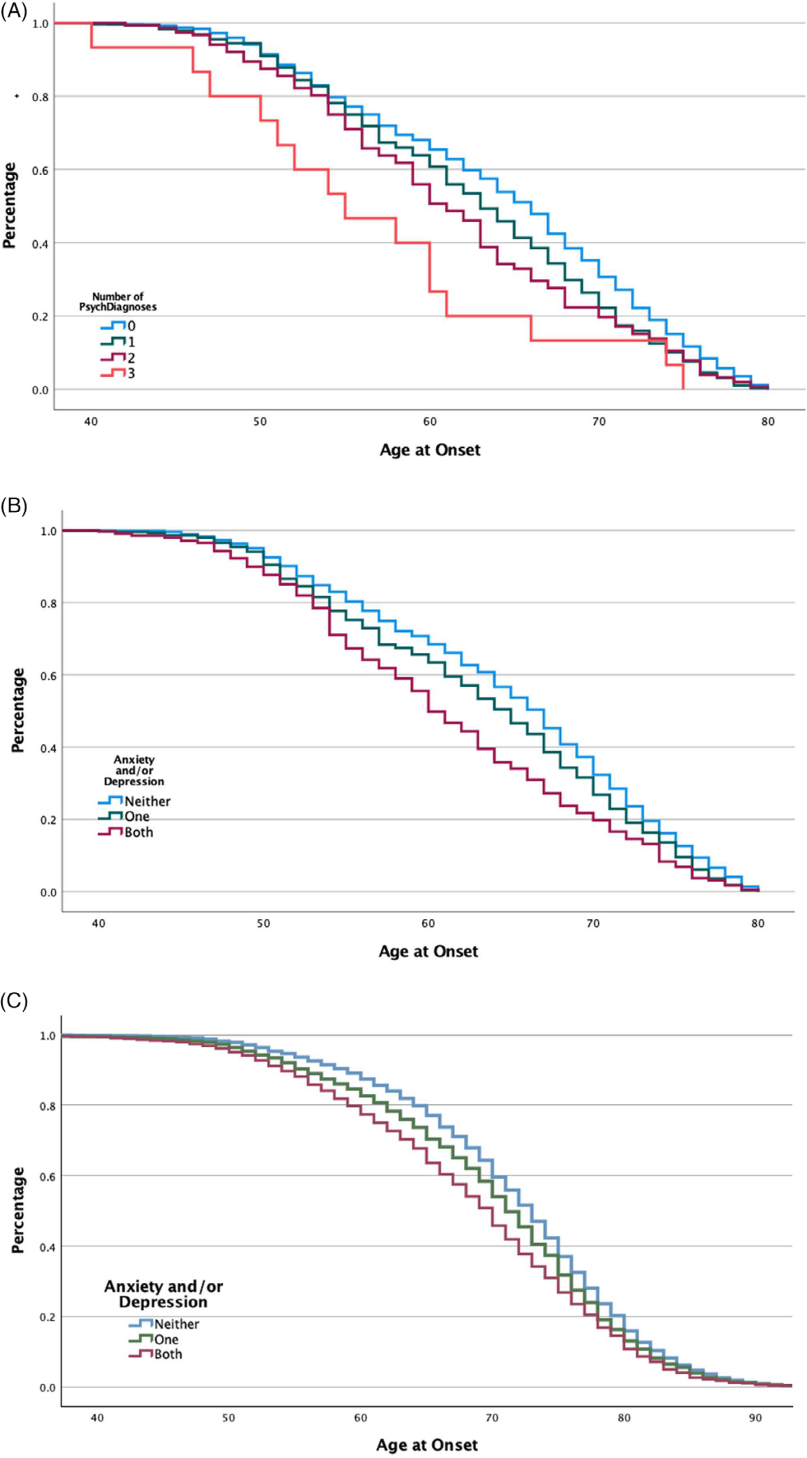
Survival Curve Burden of Psychiatric Disorders. (A) In the UCSF MAC cohort, plotting individuals stratified by numbers of psychiatric conditions (depression, anxiety, PTSD, bipolar disorder, and schizophrenia) versus the age at which they developed first symptoms of Alzheimer's disease produced four distinct Kaplan‐Meier curves. The blue line (*n* = 705) encompasses all participants who lacked any psychiatric condition. The green line (*n* = 436) included only those who had one psychiatric condition. The red line (*n* = 337) comprised those individuals who possessed two psychiatric conditions. The orange line (*n* = 22) included those who had three or more psychiatric conditions. The exact numerical breakdown for each psychiatric condition per curve can be found in supplemental material (Supplemental Table 6a). The age at which 50% of individuals with no psychiatric conditions first developed symptoms of Alzheimer's disease was 64.3 years old, with only one psychiatric condition was 62.7 years, for two psychiatric conditions 61.4 years, and for three or more psychiatric conditions 56.6 years (*p* < 0.001). (B) In the UCSF MAC cohort, plotting individuals stratified by numbers of psychiatric conditions, limited only to depression and anxiety, versus the age at which they developed first symptoms of Alzheimer's disease produced three distinct Kaplan‐Meier curves. The blue line (*n* = 712) encompasses all participants who lacked depression and anxiety. The green line (*n* = 439) included only those who had one psychiatric condition (either depression or anxiety). The red line (*n* = 349) comprised those individuals who possessed both depression and anxiety. The age at which 50% of individuals with no psychiatric conditions first developed symptoms of Alzheimer's disease was 65.0 years old, with either depression or anxiety was 63.5 years, and for both depression and anxiety 61.2 years (*p* < 0.001). (C) In the NACC cohort via NPI‐Q, plotting individuals stratified by numbers of psychiatric conditions, limited only to depression and anxiety, versus the age at which they developed first symptoms of Alzheimer's disease produced three distinct Kaplan‐Meier curves. The exact numerical breakdown for depression and anxiety can be found in supplemental material (Supplemental Table 6b). The blue line (*n* = 3603) encompasses all participants who lacked depression and anxiety. The red line (*n* = 2830) included only those who had one psychiatric condition (either depression or anxiety). The teal line (*n* = 1834) comprised those individuals who possessed both depression and anxiety. The age at which 50% of individuals with no psychiatric conditions first developed symptoms of Alzheimer's disease was 71.7 years old, with either depression or anxiety was 70.1 years, and for both depression and anxiety 68.5 years (*p* < 0.001).

To investigate cumulative effects of individual psychiatric disorders, AD age at onset was compared between groups of AD without any psychiatric conditions to those with only one, two, or three or more diagnoses. Within the UCSF MAC cohort, the presence of one psychiatric condition was associated with a 1.5‐year younger AD age at onset (*p *< 0.001), history of two psychiatric conditions led to a 3.3 year decrease in age at onset (*p *< 0.001), and three or more conditions produced a 7.7 year reduction in age at onset (*p *< 0.001) compared to those without any psychiatric conditions (Supplemental Table 6a). Within the NACC cohort via the NPI‐Q, only ratings of depression and anxiety were available. Those with only one psychiatric condition (either depression only or anxiety only) were 1.5 years younger and those with both were 3.2 years younger than those without either depression or anxiety (each, *p *< 0.001) (Supplemental Table 6b). Within the UCSF MAC cohort, a Kaplan‐Meier curve demonstrated that the age at which 50% of those with one psychiatric condition first developed symptoms of AD was 1.6 years younger than those without any psychiatric condition, 2.9 years younger for those with two psychiatric conditions compared to none, and 7.7 years younger for those with three psychiatric conditions compared to none (Figure 2a). To compare plots between the UCSF MAC and NACC, we recreated the Kaplan‐Meier curve limited to depression and anxiety. Both cohorts were congruent in that those with depression or anxiety were younger at age at onset of AD than those without either (1.5 vs 1.6 years younger for the UCSF MAC and NACC cohorts, respectively), and those with both depression and anxiety were even younger than those with depression or anxiety only (2.3 vs 1.6 years younger) (Figure 2b & 2c). Within the NACC, using the NPI‐Q, we stratified depression and anxiety by symptom severity, which revealed that increased symptom severity also produced an inverse relationship with AD age at onset (Table 3).

**TABLE 3:** Interactions between Depression and Anxiety on AD Age at Onset in NACC on NPI‐Q.

NACC NPI‐Q	No ANX	Mild ANX	Moderate ANX	Severe ANX
**No DPN**				
Average years ± Std	71.73 ± 9.31	70.45 ± 9.19	69.65 ± 9.99	69.60 ± 10.30
(*n*)	(3603)	(874)	(394)	(82)
**Mild DPN**				
Average years ± Std	70.19 ± 10.12	69.01 ± 10.43	68.49 ± 10.20	70.02 ± 8.43
(*n*)	(1010)	(681)	(255)	(52)
**Moderate DPN**				
Average years ± Std	70.17 ± 10.64	69.02 ± 10.21	68.00 ± 10.69	67.01 ± 9.85
(*n*)	(404)	(221)	(348)	(108)
**Severe DPN**				
Average years ± Std	68.50 ± 12.10	68.77 ± 9.30	67.15 ± 10.53	67.14 ± 10.05
(*n*)	(66)	(31)	(53)	(85)

Abbreviations: AD, Alzheimer's disease; ANX, Anxiety; DPN, Depression; NACC, National Alzheimer's Coordinating Center; NPI, Neuropsychiatric Index Questionnaire.

## 4 DISCUSSION

Detailing the prevalence of major psychiatric disorders via retrospective chart review across 1,500 MAC AD participants, we found inverse associations between age at AD symptom onset and histories of depression, anxiety, and PTSD, that combined, led to even further reductions in AD age at onset. Redemonstrating these associations in over 8,000 AD participants from the NACC using the NPI‐Q, we also found that the severity of endorsed depression and anxiety symptoms inversely associated with AD age at onset. Overall, our findings suggest that the number and severity of psychiatric disorders constitute an overall burden of psychiatric disease that influences AD presentation, with wide‐ranging implications for treatment and care, discussed below.

Rates of depression (43.3%) and anxiety (32.2%) in the MAC cohort were similar to those observed in other AD cohorts (30‐50%^24^, ^30^, ^31^, ^32^, ^33^, ^34^ and 30‐40%,^35^ respectively) and were significantly higher than rates in U.S. adults aged 45‐64 and 65 and older for depression (18.4% and 14.2%^36^) and anxiety (15.2% and 11.2%^37^). Despite elevations in the rates of female sex within depression and anxiety in the MAC cohort, these were in keeping with rates observed in the US general population (63.9% and 66.6% female for depression and anxiety in the MAC cohort vs. rates of 60% female for depression 60+^38^ and anxiety 18+^37^ in US adults). Notably, AD with depression or anxiety possessed significantly younger age at onset of cognitive symptoms than those without (Table 2). Binning the duration of psychiatric symptoms into a series of age at onset epochs, the decreases in AD age at onset were no different across psychiatric symptom onset periods (Supplemental Tables 4a and 4b). In contrast, the severity of psychiatric symptoms did significantly decrease the age at onset of cognitive symptoms (Table 3). Taken together, the timing and duration of depression or anxiety symptoms was not as important for AD symptom onset as their presence and degree of severity. Beyond this, when combined, the presence of anxiety and depression produced even further decreases in AD age at onset, in both the MAC cohort (Figure 2b) and the NACC cohort (Figure 2c and Table 3), suggesting that depression and anxiety each uniquely contribute towards AD susceptibility.

As to how depression and anxiety relate to AD pathophysiology, this remains understudied. Disruptions of the posterior cingulate cortex (PCC) and default mode network (DMN), which are appreciated early in the pathophysiology of AD,^39^, ^40^ are also commonly observed in individuals suffering from depression and anxiety.^41^, ^42^, ^43^ Thus, we speculate that depression, anxiety, and AD converge on shared underlying anatomical substrates, where in those with longstanding history of psychiatric disease, depression and anxiety may represent a vulnerability factor towards developing AD (similar to the associations observed between developmental history and phenotypical presentation of AD^44^, ^45^), while in those who present with more recent onset of psychiatric symptoms depression and anxiety might reflect a prodrome of the neurodegenerative process. To this end, neurodevelopmental effects of *APOE ɛ4* have been described, with decreased PCC volumes and white matter integrity in infant carriers.^46^ Moreover, higher rates of neuropsychiatric symptoms (as measured by questionnaires of depression and anxiety, including the NPI) were associated with lower CSF beta‐amyloid and higher p‐tau levels in a large cohort of cognitively normal individuals,^47^ and were associated with early subcortical, and minimal cortical, neurofibrillary tangle accumulation, in a large autopsy proven AD cohort.^48^

To explore potential mechanisms underlying the distinct effects of depression and anxiety on age at AD onset, we integrated findings from this study with our broader screening of both established risk and novel AD factors (non‐right‐handedness, learning disability, autoimmunity, and seizures)^26^ and found significantly higher rates of autoimmune disease and seizure in AD with depression and anxiety, respectively (Table 2). There is substantial precedence for these findings as individuals with autoimmune disease are known to have higher rates of depression, the severity of which directly correlates to systemic inflammatory burden.^49^, ^50^, ^51^, ^52^, ^53^, ^54^ Depressive symptoms have also been found in association with changes in DMN connectivity mediated by increases in peripheral markers of inflammation,^55^, ^56^ and the same chronic systemic and neuroinflammatory changes upregulate beta‐amyloid expression and promote tau propagation.^57^, ^58^ Similarly, neuronal hyperexcitability has been shown to facilitate tau spread^59^ and individuals with a history of early life seizures show greater amounts of beta‐amyloid deposition in midlife.^60^ Individuals with seizures experience higher rates of anxiety, the degree to which also serves as a predictor of epilepsy severity,^61^, ^62^ and chronic anxiety has been shown to promote neuronal hyperexcitability in the hippocampus, which in turn exacerbates seizure susceptibility,^63^, ^64^, ^65^ facilitating AD pathophysiology. Taken together, we speculate that in some AD presentations, the presence and severity of depression and anxiety may reflect the outwards manifestations of underlying neuroinflammatory and neuronal hyperexcitability processes, which could be targeted for therapeutic benefit.

Regarding the impact of psychiatric disease burden on clinical phenotype, our main findings pertain to the inverse relationship with AD age at onset, and as such are most relevant to the categories of amnestic EOAD vs. amnestic LOAD. EOAD has been shown to possess more aggressive AD pathophysiology,^66^, ^67^ which may account in part for our observations. Nonetheless, we also find evidence in support of prior studies that observed higher rates of psychiatric symptoms within PCA than other AD presentations,^68^, ^69^ as we find greater amounts of depression along with a trend towards more anxiety in PCA compared to lvPPA and amnestic AD (Supplemental Table 1).

Despite the small sample size (*n* = 15), AD with a history of PTSD were significantly younger at AD age at onset than those without. PTSD often presents with co‐morbid depression and anxiety, and those with comorbid diagnoses have greater functional impairments,^70^, ^71^ perhaps explaining why in our study AD with PTSD possessed the youngest age at onset of all (Table 2). In contrast, there were no differences in AD age at onset in those with BPD and SCZ compared to those without. We observed a decreased prevalence of BPD (1.2%) and SCZ (0.4%) in the MAC cohort than expected for the general population (2.4% BPD^72^ and 1.0% SCZ^73^), raising the possibility that these are underrepresented in AD. To this end, associations between BPD and neurodegenerative disease are hypothesized to be more relevant to frontotemporal dementia,^74^ and in a study that showed SCZ increased risk of dementia, the conferred risk lacked clear AD association.^22^ Regardless of the sample size, the inclusion of these additional diagnoses had continued negative synergistic effect in decreasing AD age at onset, beyond the combination of depression and anxiety alone (Figure 2a), suggesting that they conferred additional effects in driving earlier AD presentations.

While retrospective chart review enables the investigation of novel associations, it suffers from inherent vulnerabilities to ascertainment bias. Here, the assignment of psychiatric diagnostic labels was based on either outside past medical history (e.g., a referring psychiatrist) or evaluating clinician history. Nonetheless, prevalence rates of psychiatric disease were remarkably consistent with previous AD studies, mitigating concerns for ascertainment bias. Though we contend that the higher amounts of psychiatric disease observed in EOAD vs. LOAD reflect differences in their underlying pathophysiological mechanisms, it is also important to note that there are generational differences between these cohorts and there have been changes in recognition and screening of specific psychiatric conditions over the period in which our participants lived. However, when broken down into quintiles, within the respective categories of EOAD and LOAD, younger cohorts possessed higher rates of depression, anxiety, and PTSD (Figure 1), suggesting that generational differences alone could not entirely account for the inverse, linear distribution in the data. Regarding the conspicuous lack of BPD and SCZ in the UCSF MAC cohort, future studies are necessary to determine if this reflects an underrepresentation in AD compared to the general population or is the result of systemic dropout (as BPD and SCZ are each associated with shortened average lifespans and increased barriers to health care access).^75^, ^76^ If systemic dropout were behind the lower rates of BPD and SCZ observed in our AD cohort, we should have also observed an inverse relationship with age at AD symptom onset instead of the observed flat association (Figure 1) supporting our assertion of true prevalence differences. Finally, this study was based on clinically diagnosed AD, and consequently, we lack confirmed amyloid pathology across all participants, however, a substantial portion of the UCSF MAC cohort (roughly 1/3rd of EOAD and 1/10th of LOAD) had confirmed amyloid positivity through PET and/or autopsy results, and thus met Core 1 criteria for AD.^77^ Given newer accessible blood‐based beta‐amyloid biomarker testing for AD and increased availability of tau‐PET scans, future studies will be able to more quickly verify our findings and facilitate correlations between the burden of psychiatric symptoms with specific underlying pathologies.

In conclusion, this study of approximately 10,000 clinically defined AD subjects highlights the significant impact of psychiatric comorbidities on the age of AD symptom onset. These findings suggest that psychiatric conditions contribute distinctively to AD susceptibility, underscoring the importance of obtaining detailed psychiatric symptom assessments in individuals with neurodegenerative disease.

## CONFLICT OF INTEREST STATEMENT

ZAM, EE, JD, SG, NR, RB, AP, MM, VS, LTG, WWS, KPR, JHK, HJR, MLGT, DCP, report no relevant disclosures. IEA reports no relevant disclosures. AE reports serving on the editorial board for the journal Alzheimer's & Dementia; serving on the executive committee for the neuromodulatory subcortical structures professional interest area of the International Society to Advance Alzheimer's Research and Treatment (ISTAART). SS reports receiving consulting fees from Acsel Health, Precision Xtract, and Techspert.io. GDR reports grants from the National Institutes of Health, Alzheimer's Association, the American College of Radiology, Rainwater Charitable Foundation, Avid Radiopharmaceuticals, Eli Lilly, GE Healthcare, Life Molecular Imaging, and Genentech, as well as personal fees from Axon Neurosciences, Genentech, Johnson & Johnson, F. Hoffman–La Roche, and GE Healthcare outside the submitted work for service on scientific advisory boards (Axon Neurosciences, Eisai, Genentech, and F. Hoffman–La Roche) and a data safety monitoring board (Johnson & Johnson). BLM reports serving on the Cambridge National Institute for Health Research Biomedical Research Centre advisory committee and its subunit, the Biomedical Research Unit in Dementia; serving as a board member for the American Brain Foundation; serving on John Douglas French Alzheimer's Foundation board of directors; serving on the Safely You board of directors; serving as scientific director for the Tau Consortium; serving as medical advisor for and receiving a grant from The Bluefield Project for Frontotemporal Dementia Research; serving as a consultant for Rainwater Charitable Foundation, Stanford Alzheimer's Disease Research Center, Buck Institute SAB, Larry L. Hillblom Foundation, University of Texas Center for Brain Health, University of Washington Alzheimer's Disease Research Center EAB, and Harvard University Alzheimer's Disease Research Center EAB; receiving royalties from Guilford Press, Cambridge University Press, Johns Hopkins Press, and Oxford University Press; serving as editor for Neurocase; serving as section editor for Frontiers in Neurology; and receiving grants P30 AG062422, P01 AG019724, R01 AG057234, and T32 AG023481 from the NIH. Author disclosures are available in the supporting information.

## CONSENT STATEMENT

Human research committees at all included study sites approved the study of patients’ clinical data. Written informed consent from participants or responsible surrogates was obtained in accordance with the Declaration of Helsinki.

## Supporting information



Supporting information

Supporting information
