# Supplementary material for: Burden of psychiatric disease inversely correlates with Alzheimer's age at onset
Source: Alzheimers Dement. 2025 Oct 23;21(10):e70677. doi: 10.1002/alz.70677 (PMC12549220; doi:10.1002/alz.70677)
Supplement: Supplementary file 1 — Supporting information [file ALZ-21-e70677-s002.docx]

**Supplemental Table 1. Psychiatric Disorders in Amnestic vs. Non-amnestic AD and lvPPA vs. PCA in UCSF MAC**

| **Group Demographics** | **Amnestic (n=1216)** | | **Non-amnestic (284)** | | ***p*** | **lvPPA**  **(n=171)** | | **PCA**  **(n=113)** | | ***p*** |
| --- | --- | --- | --- | --- | --- | --- | --- | --- | --- | --- |
| **Age at onset**  Average years ± Std | **64.6 ± 9.3** | | **59.7 ± 7.9** | | **<0.001** | **60.7 ± 8.3** | | **58.1 ± 7.1** | | **<0.01** |
| **Age at first visit**  Average years ± Std | **68.6 ± 9.0** | | **63.6 ± 7.9** | | **<0.001** | **64.5 ± 8.1** | | **62.1 ± 7.2** | | **<0.01** |
| **Sex**  % Male (n) | 43.4%  (528/1216) | | 41.2%  (117/284) | | 0.495 | 45.6%  (78/171) | | 34.5%  (39/113) | | 0.063 |
| **Education**  Average years ± Std | **15.0 ± 3.9**  **(1182)** | | **15.7 ± 2.8**  **(275)** | | **<0.01** | 15.7 ± 2.8  (164) | | 16.1 ± 2.4  (106) | | 0.780 |
| ***APOE*-ɛ4 carriers** | **61.9%**  **(193/312)** | | **44.2%**  **(65/147)** | | **<0.001** | 43.8%  (39/89) | | 44.8%  (26/58) | | 0.903 |
| **Psychiatric Disorders** |  | |  | |  |  | |  | |  |
| **Depression**  % Depression (n) | 43.9%  (534/1216) | | 41.5%  (118/284) | | 0.469 | **36.8%**  **(63/171)** | | **48.7%**  **(55/113)** | | **0.048** |
| **Anxiety**  % Anxiety (n) | 31.5%  (383/1216) | | 35.9%  (102/284) | | 0.151 | 31.6%  (54/171) | | 42.5%  (48/113) | | 0.061 |
| **PTSD**  % PTSD (n) | 1.2%  (14/1216) | | 1.1%  (3/284) | | 0.893 | 1.2%  (2/171) | | 0.1%  (1/113) | | 0.820 |
| **BPD**  % Bipolar Disorder (n) | 1.4%  (17/1216) | | 0.4%  (1/284) | | 0.145 | 0.0%  (0/171) | | 0.1%  (1/113) | | 0.218 |
| **SCZ**  % Schizophrenia (n) | 0.5%  (6/1216) | | 0.0%  (0/284) | | 0.236 | 0.0%  (0/171) | | 0.0%  (0/113) | | 1 |
| **Increased Non-amnestic/** | **95% C.I. for OR** | | | |  | **95% C.I. for OR** | | | |  |
| **PCA Prevalence** | **OR** | **Lower** | | **Upper** | ***p*** | **OR** | **Lower** | | **Upper** | ***p*** |
| **Depression** | 1.010 | 0.848 | | 1.431 | 0.469 | **1.626** | **1.004** | | **2.633** | **0.048** |
| **Anxiety** | 0.820 | 0.626 | | 1.076 | 0.152 | 1.600 | 0.977 | | 2.620 | 0.062 |
| **PTSD** | 0.934 | 0.262 | | 3.330 | 0.916 |  |  | |  |  |
| **BPD** | 4.013 | 0.532 | | 30.276 | 0.178 |  |  | |  |  |
| **SCZ** |  |  | |  |  |  |  | |  |  |

AD = Alzheimer’s disease; C.I. = confidence interval; lvPPA = logopenic variant of Primary Progressive Aphasia;

PCA = Posterior Cortical Atrophy; PTSD = Post-traumatic stress disorder; BPD = Bipolar disorder; SCZ = Schizophrenia;

OR = Odds Ratio; Std = standard deviation.

* <0.05; ** <0.001

**Supplemental Table 2. Depression and Anxiety by External Diagnosis or Internal Report**

| **Group** | **External Diagnosis of DPN** | **Internal Report of DPN** | **External Diagnosis of ANX** | **Internal Report of ANX** |
| --- | --- | --- | --- | --- |
| **Demographics** | **(n= 419)** | **(n= 233)** | **(n= 213)** | **(n= 272)** |
| **Age at onset**  Avg yrs ± Std | 62.1 ± 9.0 | 62.9 ± 9.6 | 61.0 ± 9.1 | 62.0 ± 9.4 |
| **Age at first visit**  Avg yrs ± Std | 66.3 ± 9.5 | 66.9 ± 9.2 | 65.0 ± 8.7 | 66.0 ± 9.1 |

Dx = Diagnosis; DPN = Depression; ANX = Anxiety

**Supplemental Table 3. Depression and Anxiety and AD Age at Onset in NACC on NPI-Q**

|  | **No DPN** | **DPN** | **No ANX** | **ANX** |
| --- | --- | --- | --- | --- |
|  | **(n=4953)** | **(n=3314)** | **(n=5083)** | **(n=3184)** |
| **Age at onset**  Avg yrs ± Std | **71.3 ± 9.4**** | **69.2 ± 10.4**** | **71.3 ± 9.7**** | **69.2 ±10**** |

AD = Alzheimer’s disease; NACC = National Alzheimer's Coordinating Center;

NPI-Q = Neuropsychiatric Index Questionnaire; DPN = Depression; ANX = Anxiety

** <0.001

**Supplemental Table 4a. Depression by Years Prior to Onset of AD**

|  |  |  | **DPN Pre** |  |  | **DPN Pre** | **DPN Post** | **Unknown** |  |
| --- | --- | --- | --- | --- | --- | --- | --- | --- | --- |
| **Group** | **>40** | **31-40** | **21-30yrs** | **11-20yrs** | **0-10yrs** | **Total** | **1^st^ AD sx** | **Duration** | **No DPN** |
| **Demographics** | **(n=56)** | **(n=20)** | **(n=26)** | **(n=39)** | **(n=112)** | **(n=253)** | **(n=287)** | **(n=111)** | **(n=848)** |
| **Age at onset**  Average years ± Std | 61.1 ± 9.5 | 61.8 ± 7.5 | 61.5 ± 8.0 | 62.9 ± 9.7 | 62.1 ± 9.2 | 61.2 ± 9.1 | 62.4 ± 9.7 | 63.6 ± 8.0 | 64.6 ± 9.2 |
| **Age at first visit**  Average years ± Std | 65.8 ± 9.3 | 66.5 ± 7.9 | 65.2 ± 7.2 | 67.8 ± 9.4 | 65.9 ± 9.0 | 66.2 ± 8.8 | 66.4 ± 9.3 | 67.5 ± 7.7 | 68.5 ± 9.1 |
| **Sex**  % Male (n) | 28.6% (16/56) | 30.0%  (6/20) | 34.5%  (10/26) | 48.7% (19/39) | 33.9% (38/112) | 34.8% (88/253) | 41.5%  (119/287) | 26.1%  (29/111) | 48.4% (409/848) |
| **Typical Risk Factors** |  |  |  |  |  |  |  |  |  |
| **Education**  Average years ± Std | 15.4 ± 3.1  (51) | 16.4 ± 2.7  (20) | 15.3 ± 5.2  (24) | 14.9 ± 5.0 (39) | 14.9 ± 3.2  (106) | 15.2 ± 3.7  (241) | 14.8 ± 3.7  (283) | 15.4 ± 4.2  (106) | 15.2 ± 3.6  (825) |
| ***APOE*-ɛ4 carriers** | 63.6%  (7/11) | 0.0%  (0/2) | 77.8%  (7/9) | 36.4%  (4/11) | 66.7%  (26/39) | 62.0% (44/71) | 53.8%  (50/93) | 66.7%  (24/36) | 54.1% (140/259) |
| **Hypertension**  % HTN (n) | 44.6%  (25/56) | 25.0%  (5/25) | 34.6%  (9/26) | 48.7% (19/39) | 42.9%  (48/112) | 42.0% (106/253) | 41.1%  (118/287) | 39.6%  (44/111) | 47.5% (403/848) |
| **Hyperlipidemia**  % HLD (n) | 42.9% (24/56) | 35.0%  (7/20) | 30.8%  (8/26) | 41.0% (16/39) | 47.3%  (53/112) | 42.7% (108/253) | 47.4%  (136/287) | 44.1%  (49/111) | 50.8% (431/848) |
| **Diabetes**  % DM (n) | 8.9% (5/56) | 5.0%  (1/20) | 11.5%  (3/26) | 10.2%  (4/39) | 10.7%  (12/112) | 9.9% (25/253) | 6.6%  (19/287) | 13.5%  (15/111) | 12.4% (105/848) |
| **Novel Factors** |  |  |  |  |  |  |  |  |  |
| **non-Right-Handed**  % nRH (n) | 12.5%  (7/56) | 20.0%  (4/20) | 11.5%  (3/26) | 23.1% (9/39) | 8.9%  (10/112) | 13.0% (33/253) | 10.1%  (29/287) | 10.8%  (12/111) | 12.3%  (104/848) |
| **Learning Disability**  % LD (n) | 12.5%  (7/56) | 20.0%  (4/20) | 3.8%  (1/26) | 7.7% (3/39) | 6.3%  (7/112) | 8.7% (22/253) | 6.6%  (19/287) | 9.0%  (10/111) | 5.9% (50/848) |
| **Seizure**  % Seizure (n) | 12.5% (7/56) | 15.0%  (3/20) | 0.0%  (0/26) | 7.7%  (3/39) | 7.1%  (8/112) | 8.3% (21/253) | 6.6%  (19/287) | 7.2%  (8/111) | 10.7% (52/848) |
| **Autoimmune Disease**  % Autoimmunity (n) | 35.7% (20/56) | 40.0%  (8/20) | 15.4%  (4/26) | 25.6% (10/39) | 18.8%  (21/112) | 25.7% (65/253) | 26.5%  (76/287) | 23.4%  (26/111) | 20.2%  (171/848) |
| **Psych Dz** |  |  |  |  |  |  |  |  |  |
| **Depression**  % Depression (n) |  |  |  |  |  |  |  |  |  |
| **Anxiety**  % Anxiety (n) | 66.1%  (37/56) | 55.0%  (11/20) | 42.3%  (11/26) | 61.5%  (24/39) | 60.7%  (68/112) | 59.7%  (151/253) | 49.8%  (143/287) | 49.6%  (55/111) | 16.1%  (136/848) |
| **PTSD**  % PTSD (n) | 10.7%  (6/56) | 5.0%  (1/20) | 3.8%  (1/26) | 0.0%  (0/39) | 1.8%  (2/112) | 4.0%  (10/253) | 0.7%  (2/287) | 2.7%  (3/111) | 0.1%  (1/848) |
| **Bipolar Disorder**  % BPD (n) | 5.4%  (3/56) | 5.0%  (1/20) | 11.5%  (3/26) | 7.7% (3/39) | 1.8%  (2/112) | 4.7%  (12/253) | 0.0%  (0/287) | 1.8%  (2/111) | 0.5%  (4/848) |
| **Schizophrenia**  % Schizophrenia (n) | 1.8%  (1/56) | 0.0%  (0/20) | 3.8%  (1/26) | 0.0%  (0/39) | 0.0%  (0/112) | 0.8%  (2/253) | 0.3%  (1/287) | 0.0%  (0/111) | 0.4%  (3/848) |

AD = Alzheimer’s disease; DPN = Depression; PTSD = Post-traumatic stress disorder; BPD = Bipolar disorder; SCZ = Schizophrenia; Std = standard deviation.

**Supplemental Table 4b. Anxiety by Years Prior to Onset of AD**

|  |  |  | **ANX Pre** |  |  | **ANX Pre** | **ANX Post** | **Unknown** |  |
| --- | --- | --- | --- | --- | --- | --- | --- | --- | --- |
| **Group** | **>40** | **31-40** | **21-30yrs** | **11-20yrs** | **0-10yrs** | **Total** | **1^st^ AD sx** | **Duration** | **No ANX** |
| **Demographics** | **(n=36)** | **(n=10)** | **(n=9)** | **(n=21)** | **(n=67)** | **(n=143)** | **(n=282)** | **(n=58)** | **(n=1015)** |
| **Age at onset**  Average years ± Std | 63.6 ± 9.1 | 60.4 ± 10.0 | 61.4 ± 9.4 | 63.0 ± 9.3 | 61.1 ± 9.9 | 62.0 ± 9.5 | 61.5 ± 10.1 | 61.1 ± 9.3 | 64.6 ± 9.1 |
| **Age at first visit**  Average years ± Std | 67.8 ± 8.6 | 64.0 ± 8.8 | 65.8 ± 9.3 | 67.5 ± 8.1 | 64.8 ± 9.6 | 65.9 ± 9.1 | 65.4 ± 8.8 | 65.1 ± 9.2 | 68.6 ± 8.9 |
| **Sex**  % Male (n) | 27.8% (10/36) | 10.0%  (1/10) | 22.2%  (2/9) | 57.1% (12/21) | 25.4% (17/67) | 29.4%  (42/143) | 36.9% (104/282) | 25.9%  (15/58) | 47.6% (483/1015) |
| **Typical Risk Factors** |  |  |  |  |  |  |  |  |  |
| **Education**  Average years ± Std | 15.7 ± 2.8  (34) | 15.0 ± 2.0  (10) | 13.4 ± 5.5  (8) | 16.1 ± 3.7 (20) | 15.7 ± 3.4  (61) | 15.6 ± 3.4  (133) | 15.1 ± 3.6  (270) | 15.5 ± 3.1 (56) | 15.1 ± 3.8 (990) |
| ***APOE*-ɛ4 carriers** | 50.0%  (2/4) | 0.0%  (0/1) | 100%  (2/2) | 50.0%  (3/6) | 64.0%  (16/25) | 60.5%  (23/38) | 51.6%  (49/95) | 60.0%  (9/15) | 56.9%  (177/311) |
| **Hypertension**  % HTN (n) | 50.0%  (18/36) | 30.0%  (3/10) | 55.6%  (5/9) | 42.9% (9/21) | 34.3%  (23/67) | 40.6%  (58/143) | 38.3%  (108/282) | 44.8%  (26/58) | 47.2% (479/1015) |
| **Hyperlipidemia**  % HLD (n) | 33.3% (12/36) | 10.0%  (1/10) | 11.1%  (1/9) | 61.9% (13/21) | 50.7%  (34/67) | 42.7%  (61/143) | 43.3%  (122/282) | 43.1%  (25/58) | 50.9% (517/1015) |
| **Diabetes**  % DM (n) | 5.6% (2/36) | 0.0%  (0/10) | 0.0%  (0/9) | 9.5%  (2/21) | 7.5%  (5/67) | 6.3%  (9/143) | 5.7%  (16/282) | 12.1%  (7/58) | 13.0% (132/1015) |
| **Novel Factors** |  |  |  |  |  |  |  |  |  |
| **non-Right-Handed**  % nRH (n) | 13.6%  (5/36) | 10.0%  (1/10) | 11.1%  (1/9) | 23.8% (5/21) | 10.4%  (7/67) | 13.3%  (19/143) | 11.7%  (33/282) | 8.6%  (5/58) | 12.0% (122/1015) |
| **Learning Disability**  % LD (n) | 11.1%  (4/36) | 20.0%  (2/10) | 0.0%  (0/9) | 19.0% (4/21) | 3.0%  (2/67) | 8.4%  (12/143) | 8.8%  (25/282) | 5.2%  (3/58) | 6.0% (61/1015) |
| **Seizure**  % Seizure (n) | 11.1% (4/36) | 20.0%  (2/10) | 0.0%  (0/10) | 9.5%  (2/21) | 10.4%  (7/67) | 10.5%  (15/143) | 9.2%  (26/282) | 8.6%  (5/58) | 5.3% (54/1015) |
| **Autoimmune Disease**  % Autoimmunity (n) | 30.6% (11/36) | 20.0%  (2/10) | 11.1%  (1/9) | 23.8% (5/21) | 23.9%  (16/67) | 24.5%  (35/143) | 23.0%  (65/282) | 20.7%  (12/58) | 22.3% (226/1015) |
| **Psych Dz** |  |  |  |  |  |  |  |  |  |
| **Depression**  % Depression (n) | 75.5%  (27/36) | 100.0%  (10/10) | 88.9%  (8/9) | 66.7%  (14/21) | 80.6%  (54/67) | 79.0%  (113/143) | 67.0%  (189/282) | 77.6%  (45/58) | 30.0% (304/1015) |
| **Anxiety**  % Anxiety (n) |  |  |  |  |  |  |  |  |  |
| **PTSD**  % PTSD (n) | 25.0%  (9/36) | 10.0%  (1/10) | 22.2%  (2/9) | 0.0%  (0/21) | 3.0%  (2/67) | 9.8%  (14/143) | 0.3%  (1/282) | 3.4%  (2/58) | 0.0%  (0/1015) |
| **Bipolar Disorder**  % BPD (n) | 0.0%  (0/36) | 0.0%  (0/10) | 0.0%  (0/9) | 0.0%  (0/21) | 1.5%  (1/67) | 0.7%  (1/143) | 1.7%  (5/282) | 3.4%  (2/58) | 1.0%  (10/1015) |
| **Schizophrenia**  % Schizophrenia (n) | 2.8%  (1/36) | 0.0%  (0/10) | 0.0%  (0/9) | 0.0%  (0/21) | 0.0%  (0/67) | 0.7%  (1/143) | 0.3%  (1/282) | 0.0%  (0/58) | 0.4%  (4/1015) |

AD = Alzheimer’s disease; ANX = Anxiety; DPN = Depression; PTSD = Post-traumatic stress disorder; BPD = Bipolar disorder; SCZ = Schizophrenia; OR = odds ratio; Std = standard deviation.

**Supplemental Table 5a. UCSF MAC AD Cohort Broken into Quintiles**

|  | **UCSF MAC 1,500 AD** | | | | |
| --- | --- | --- | --- | --- | --- |
| **Group** | **1** | **2** | **3** | **4** | **5** |
| **Demographics** | **(n=300)** | **(n=300)** | **(n=300)** | **(n=300)** | **(n=300)** |
| **Age at onset**  Average years ± Std | **50.2 ± 3.1**** | **57.7 ± 2.2**** | **64.6 ± 1.8**** | **69.9 ± 1.6**** | **75.9 ± 2.1**** |
| **Age at first visit**  Average years ± Std | **54.9 ± 3.9**** | **62.0 ± 3.6**** | **68.7 ± 3.0**** | **73.5 ± 2.8**** | **78.9 ± 2.5**** |
| **Sex**  % Male (n) | 40% (120/300) | 42.7% (128/300) | 46.7% (140/300) | 43.7% (131/300) | 42% (126/300) |
| **Education**  Average years ± Std | 15 ± 3.2 (295) | 15.3 ± 3.7 (289) | 15.3 ± 4.1 (289) | 15.2 ± 3.8 (291) | 15 ± 3.6 (293) |
| ***APOE*-ɛ4 carriers** | 52.2%  (70/134) | 52.1% (62/119) | 60.5%  (49/81) | 61.9%  (39/63) | 61.3%  (38/62) |
| **Psych Dz** |  |  |  |  |  |
| **Depression**  % Depression (n) | **51.0%** (153/300)** | **49.3%** (148/300)** | **43.3%** (130/300)** | **37.7%** (113/300)** | **36.3%** (109/300)** |
| **Anxiety**  % Anxiety (n) | **46.7%** (122/300)** | **41.7%** (125/300)** | **30.3%** (91/300)** | **23.7%** (71/300)** | **25.3%** (76/300)** |
| **PTSD**  % PTSD (n) | 2.3% (7/300) | 1.0% (3/300) | 0.7% (2/300) | 0% (0/300) | 1.0% (3/300) |
| **BPD**  % Bipolar Disorder (n) | 0.7% (2/300) | 2.3% (7/300) | 0.3% (1/300) | 1.7% (5/300) | 0.3% (1/300) |
| **SCZ**  % Schizophrenia (n) | 0.7% (2/300) | 0.7% (2/300) | 0% (0/300) | 0.7% (2/300) | 0% (0/300) |

AD = Alzheimer’s disease; PTSD = Post-traumatic stress disorder; BPD = Bipolar disorder; SCZ

= Schizophrenia; Std = standard deviation.

* <0.05; ** <0.001

**Supplemental Table 5b. NACC Cohort Broken into Quintiles**

|  |  | | | | |
| --- | --- | --- | --- | --- | --- |
| **Group** | **1** | **2** | **3** | **4** | **5** |
| **Demographics** | **(n=1653)** | **(n=1653)** | **(n=1653)** | **(n=1653)** | **(n=1655)** |
| **Age at onset**  Average years ± Std | **55.5 ± 5.4**** | **66.1 ± 2.0**** | **71.6 ± 1.4**** | **76.3 ± 1.4**** | **82.9 ± 3.6**** |
| **Age at first visit**  Average years ± Std | **60.9 ± 6.4**** | **71.4 ± 4.0**** | **76.7 ± 3.4**** | **80.7 ± 3.0**** | **86.5 ± 4.0**** |
| **Sex**  % Male (n) | **47.7%** (789/1653** | **44.1%** (730/1653)** | **44.4%** (734/1653)** | **41.7%** (687/1653)** | **40.6%** (672/1655)** |
| **Education**  Average years ± Std | **14.5 ± 3.6** (1642)** | **14.2 ± 3.7** (1646)** | **13.9 ± 3.9** (1644)** | **13.8 ± 3.8** (1640)** | **13.3 ± 4.1** (1648)** |
| ***APOE*-ɛ4 carriers** | **58.7%****  **(734/1250)** | **71.4%** (857/1200)** | **64.4%****  **(769/1194)** | **56.7%****  **(668/1179)** | **42.2%****  **(466/1105)** |
| **Psych Dz** |  |  |  |  |  |
| **Depression**  % Depression (n) | **48.9%** (809/1653)** | **41.8%** (691/1653)** | **39.1%** (645/1653)** | **35.8%** (592/1653)** | **35.2%** (583/1655)** |
| **Anxiety**  % Anxiety (n) | **46.7%** (772/1653)** | **41.0%** (676/1653)** | **37.7%** (624/1653)** | **34.1%** (564/1653)** | **32.8%** (543/1655)** |
| **PTSD**  % PTSD (n) |  |  |  |  |  |
| **BPD**  % Bipolar Disorder (n) |  |  |  |  |  |
| **SCZ**  % Schizophrenia (n) |  |  |  |  |  |

AD = Alzheimer’s disease; PTSD = Post-traumatic stress disorder; BPD = Bipolar disorder; SCZ

= Schizophrenia; Std = standard deviation.

* <0.05; ** <0.001

**Supplemental Table 6a. UCSF MAC AD and Burden of Psychiatric Disorders**

| **Group** | **No Psych Disorder** | **1 Psych Disorder** | **2 Psych Disorders** | **3+ Psych Disorders** |
| --- | --- | --- | --- | --- |
| **Demographics** | **(n=705)** | **(n=436)** | **(n=337)** | **(n=22)** |
| **Age at onset**  Average years ± Std | **64.9 ± 9.1**** | **63.4 ± 9.2**** | **61.6 ± 9.1**** | **57.2 ± 9.3**** |
| **Age at first visit**  Average years ± Std | **68.8± 9.0**** | **67.5 ± 9.0**** | **65.6 ± 8.8**** | **61.7 ± 8.0**** |
| **Sex**  % Male (n) | **50.8%** (358/705)** | **38.5%****  **(168/436)** | **32.4%** (110/339)** | **40.1%** (9/22)** |
| **Typical Risk Factors** |  |  |  |  |
| **Education**  Average years ± Std | 15.2 ± 3.7  (688) | 15.2 ± 3.6  (420) | 15.2 ± 3.6  (325) | 15.8 ± 3.1  (253) |
| ***APOE*-ɛ4 carriers** | 54.8%  (120/219) | 58.0%  (76/131) | 58.7%  (61/104) | 20%  (1/5) |
| **Hypertension**  % HTN (n) | **49.4%***  **(348/705)** | **41.3%***  **(180/436)** | **39.8%***  **(134/337)** | **45.5%* (10/22)** |
| **Hyperlipidemia**  % HLD (n) | **52.2%* (368/705)** | **46.8%***  **(204/436)** | **43.3%***  **(146/337)** | **31.8%* (7/22)** |
| **Diabetes**  % DM (n) | **13.9%***  **(98/705)** | **9.2%***  **(40/436)** | **7.4%***  **(25/337)** | **4.5%***  **(1/22)** |
| **Novel Factors** |  |  |  |  |
| **non-Right-Handed**  % nRH (n) | 12.6%  (89/705) | 10.8%  (47/436) | 11.6%  (39/337) | 18.1%  (4/22) |
| **Learning Disability**  % LD (n) | 5.8% (41/705) | 6.7%  (29/436) | 8.3%  (28/337) | 13.6%  (3/22) |
| **Seizure**  % Seizure (n) | 5.4% (38/705) | 6.9%  (30/436) | 8.9%  (30/337) | 9.1%  (2/22) |
| **Autoimmune Disease**  % Autoimmunity (n) | 20.6% (145/705) | 23.4%  (102/436) | 25.5%  (86/337) | 22.7% (5/22) |
| **Psych Dz** |  |  |  |  |
| **Depression**  % Depression (n) | 0.0%  (0/705) | 67.9%  (296/436) | 99.4%  (335/337) | 100%  (22/22) |
| **Anxiety**  % Anxiety (n) | 0.0%  (0/705) | 30.7%  (134/436) | 97.6%  (329/337) | 100%  (22/22) |
| **PTSD**  % PTSD (n) | 0.0% (0/705) | 0.0%  (0/436) | 0.3%  (1/337) | 63.6%  (14/22) |
| **BPD**  % Bipolar Disorder (n) | 0.0% (0/705) | 0.7%  (3/436) | 2.4%  (8/337) | 31.8%  (7/22) |
| **SCZ**  % Schizophrenia (n) | 0.0% (0/705) | 0.7%  (3/436) | 0.3%  (1/337) | 9.1%  (2/22) |

AD = Alzheimer’s disease; PTSD = Post-traumatic stress disorder; BPD = Bipolar disorder;

SCZ = Schizophrenia; Std = standard deviation.

* <0.05; ** <0.001

**Supplemental Table 6b. Depression and Anxiety on AD Age at Onset in NACC on NPI-Q**

| **NACC NPI-Q** | **No Psych Dz** | **Depression only** | **Anxiety only** | **Depression & Anxiety** |
| --- | --- | --- | --- | --- |
| **Age at onset**  Average years ± Std | **71.7** ± **9.3****  (3603) | **70.1** ± **10.4****  (1480) | **70.2** ± **9.5****  (1350) | **68.5** ± **10.3****  (1834) |

AD = Alzheimer’s disease; NACC = National Alzheimer's Coordinating Center; NPI-Q = Neuropsychiatric

Index Questionnaire

* <0.05; ** <0.001
